# Caregiver-reported quality of life in individuals with developmental and epileptic encephalopathy and other severe neurodevelopmental encephalopathies

**DOI:** 10.1007/s11136-025-04153-0

**Published:** 2026-01-14

**Authors:** Natasha N. Ludwig, Melissa K. Licari, Mary Wojnaroski, Gabrielle Conecker, JayEtta Hecker, Rebecca Hommer, Kelly Muzyczka, Peter Jacoby, Jenny Downs

**Affiliations:** 1 Kennedy Krieger Institute, Johns Hopkins School of Medicine, Center for Neuropsychological and Psychological Assessment/Psychiatry and Behavioral Sciences, Baltimore, MD USA; 2 The Kids Research Institute Australia, Centre for Child Health Research, The University of Western Australia, PO Box 855, West Perth, WA 6872 Australia; 3 Department of Psychology/Psychiatry and Behavioral Health, Nationwide Children’s Hospital, Ohio State University, Columbus, OH USA; 4 The Inchstone Project, Decoding Developmental Epilepsies, Washington, DC USA; 5 Connections Beyond Sight and Sound Maryland & DC DeafBlind Project, University of Maryland, College Park, MD USA; 6 Curtin School of Allied Health, Curtin University, Perth, Australia

**Keywords:** Developmental and epileptic encephalopathy, Functional abilities, Participation, Quality of life, Regression trees

## Abstract

**Purpose:**

Information on factors contributing to quality of life (QOL) informs meaningful patient-centred care. We evaluated factors influencing QOL in individuals with developmental and epileptic encephalopathy (DEE) and other severe neurodevelopmental encephalopathy conditions using hypothesis-free regression tree analysis.

**Methods:**

A questionnaire was completed by 242 caregivers of individuals two years or older. QOL was measured using the Quality of Life Inventory-Disability (QI-Disability). Independent variables described health, functional abilities and daily activities. The R package *rpart* was used to build the regression trees to explore the most influential factors associated with QOL.

**Results:**

Median age was 8.8y (interquartile range 4.6–14.9 y). Mean total QI-Disability score was 60.2 ± 14.1 out of a total possible score of 100. The subgroup with the lowest QOL scores comprised individuals with low (raw score < 4) cognition scores measured with the Developmental Profile-4 (*n* = 52, mean score 46.4) whereas higher QOL scores were achieved by individuals with higher cognition scores and capacity to engage actively when using a touchscreen (*n* = 123, mean score 67.5).

**Conclusion:**

Regression tree analysis suggests that cognition and use of touchscreens were important factors for QOL. Findings suggest small neurodevelopmental and functional gains may meaningfully improve quality of life for individuals with severe neurodevelopmental encephalopathy.

## Abstract (plain language summary)

People with severe neurodevelopmental conditions experience challenges that affect their everyday lives, including difficulties with health, functional abilities and independence in activities of daily living. There is limited understanding of what impacts quality of life in this population. In this study, caregivers of 242 individuals with severe neurodevelopmental conditions completed a questionnaire to capture the child’s quality of life, health, everyday functioning and daily activities. The analysis searched for the factors from other measures administered that were important in predicting quality of life. The most important domain predicting the total quality of life score was cognition. Individuals with lower cognition scores had lower quality of life scores while those with higher cognition scores, particularly those with ability to participate in touchscreen activities, had higher quality of life scores. Findings suggest the value of monitoring and supporting even small functional gains in alertness and hand function skills to enable more engagement with people and objects may meaningfully improve quality of life.

## Introduction

Individuals with severe neurodevelopmental encephalopathy conditions live with profoundly impacted functional impairments along with co-occurring medical conditions [1]. Within this group, there are many developmental and epileptic encephalopathy (DEE) conditions that are rare, severe, and complex and usually present early in life [2; 3]. Many DEEs have a genetic basis, although in some cases, individuals present with symptoms consistent with DEEs but without an identified genetic cause [4, 5]. Despite variability in genetic origins and clinical presentation, characteristics usually include seizures and severe to profound impairment in neurodevelopmental outcomes [3].

The range of neurological and co-occurring conditions includes motor disorders, atypical tone, orthopaedic conditions, gastrointestinal problems and cortical vision impairment (CVI). Cognitive abilities are often severely impacted and a diagnosis of intellectual developmental disorder is common. Many display symptoms of other neurodevelopmental disorders including autism spectrum disorder, attention deficit hyperactivity disorder and other behavioural disorders which further impact participation in daily life [2, 3]. Functional abilities in areas beyond cognition, including mobility (e.g., walking), fine motor abilities (e.g., grasping, ability to precisely use a touchscreen), feeding and communication (e.g., indicating understanding, speaking), are also compromised [6–8]. Limitations in functional skills can hinder participation in daily life and restrict progress toward functional independence. These co-occurring conditions complicate treatment and increase substantially the physical, emotional and caregiving burdens on families [9].

Quality of life (QOL) is increasingly recognised as a central outcome in the assessment and management of neurodevelopmental conditions [10, 11]. QOL encompasses a broad range of life domains, including health, emotional wellbeing and social participation [12, 13]. In individuals with DEE and other severe neurodevelopmental encephalopathies, difficult to manage seizures, the impacts of other comorbidities, functional impairments and reduced capacity for independence can each impact QOL [14, 15]. However, the clinical and functional factors that most strongly influence QOL in this population are poorly understood. This knowledge is critical for informing effective, meaningful and patient-focused interventions that have potential for improving QOL for this underserved population.

In this study, we used regression tree modelling to examine the contribution of clinical, neurodevelopmental, functional and participation-related factors to QOL, as reported by parents in a large community survey. By exploring how these factors interact to influence QOL, we aimed to identify key factors that could guide more targeted approaches to support individuals with DEE and other severe neurodevelopmental encephalopathies and improve their QOL.

## Method

### Study design

This was a cross-sectional observational study conducted using an online survey led by The Inchstone Project, a multistakeholder team science initiative that aims to identify and develop clinical outcome assessments validated for individuals with severe neurodevelopmental encephalopathies [16]. The Inchstone core research team includes caregivers, clinicians, a teacher, and scientists. All Inchstone research activities are guided by a Steering Committee comprised of additional caregivers, clinicians and scientists as well as an Industry Advisory Board. Importantly, individuals with lived experience were actively involved in all aspects of study design, conduct, interpretation and dissemination.

### Survey participants

Convenience sampling was employed. Parent advocacy groups (PAGs) from various DEE communities within the DEE-P Connections network (https://deepconnections.net/) invited their members to participate in an online survey, administered via CLIRINX^®^ [17] between June and November 2023. DEE-P Connections is a consortium of 50 PAGs representing patients with DEEs. In order to reduce the likelihood of data from unintended respondents, each PAG was given an access code unique to their group and were asked only to share the opportunity on their closed listserv rather than posting on social media.

Participants were eligible if they were primary caregivers of a child ≥ 12 months old with epilepsy, ASD, developmental delay, IDD or other neurodevelopmental conditions as well as severely impaired communication per parent report on the Communication Function Classification System (CFCS; i.e., seldom communicates effectively with others who are familiar and unfamiliar). Data for individuals 24 months and older were used in this analysis [18].

The study protocol was reviewed and determined to be exempt by the North Star Ethics Review Board (protocol # NB300112), with acknowledgement from the institution review boards of the Johns Hopkins School of Medicine and Nationwide Children’s Hospital. Additionally, the study received approval from the Human Research Ethics Committee at The University of Western Australia (2019/RA/4/20/6198). Informed consent was obtained from all participants prior to their involvement.

### Variables

The larger survey included the following sections in this order: Demographics, functional abilities, rating of symptom impacts and parent perceived priorities, the Quality of Life Disability (QI-Disability), the Developmental Profile 4th Edition (DP-4), a survey on alertness and responsiveness, and a survey on cortical vision impairment. QI-Disability was the outcome variable and all available variables on comorbidities, functional abilities and independence and participation were selected as independent variables.

#### Outcome variable

QI-Disability is a 32-item, parent-report questionnaire designed to assess QOL in people with intellectual disability [19], including DEEs [20, 21]. The questionnaire evaluates QOL across six key domains: Social Interaction (7 items), Positive Emotions (4 items), Negative Emotions (7 items), Physical Health (4 items), Leisure and the Outdoors (5 items) and Independence (5 items). Responses are transformed into a scale ranging from 0 to 100, with higher scores indicating a better QOL. Domain scores are derived by averaging the scores for each item within the domain and the total score is calculated by averaging the domain scores, where higher scores indicate better QOL [19].

#### Independent variables

The independent variables included a combination of clinical, developmental and functional measures that were available and deemed by the research team to be relevant to the QOL of people with DEE and other severe neurodevelopmental encephalopathies, based on extant literature and clinical expertise.

#### *Medical Variables*

Clinical measures included epilepsy (diagnosed with epilepsy, seizures but not diagnosed with epilepsy, no seizures) and the number of anti-seizure medications (0, 1, 2, 3, 4 or more), the presence of a movement disorder (yes, no) and altered muscle tone (hypertonia, hypotonia). Likelihood of CVI was assessed based on perceived ability to see an object/face and pay attention to it (rarely/never, sometimes, usually/always), because reported inability to see and pay attention to objects/faces is considered a risk factor for CVI [22].

*Neurodevelopmental and Functional Ability Variables*: The DP-4 was used to assess functional abilities [23]. The DP-4 is a 190-item, standardized and norm-referenced parent-report questionnaire for individuals birth through 21 years of age covering five key developmental domains: physical development (37 items), cognitive development (42 items), social-emotional development (36 items), communication (34 items) and adaptive behaviors (41 items) abilities [23]. The original validation included few people with intellectual disability [23] but in 10 children with SCN2A-DEE and severe to profound cognitive impairment, we previously identified strong and positive correlations between DP-4 scores and the Bayley Scales of Infant and Toddler Development, Fourth Edition Cognitive Scale (*r* = .84) and the Vineland Scales of Adaptive Behavior, Third Edition Comprehensive Interview (*r* = .78) [24]. Raw scores were used given the high frequency of floor effects on standardized and norm-referenced measures in individuals with DEEs [7]. All domain raw scores were used in the analysis.

Additional variables for functional abilities included the Communication Function Classification System (CFCS), developed for cerebral palsy [25] and modified for DEE conditions (communicates with anyone, communicates only with people known, inconsistently or seldom communicates) [26, 27]. An additional communication item asked about the number of spoken words (no words, 1–5 words, > 5 words). Gross motor function was grouped using the Functional Mobility Scale modified for Rett syndrome [28]. Hand function was grouped as able to use a pincer grasp, reach and grasp hand-sized objects, grasp an object if placed in the hand, or unable to grasp objects [29]. The Eating and Drinking Ability Classification System (EDACS) which was developed for cerebral palsy [25] and modified for DEE conditions was used to evaluate eating (eat and drinks; safely, some mess, some limitations/safety concerns, significant limitations/safety concerns, G-tube only) [26, 27].

#### *Everyday activities of daily living*

Everyday activities described in the Self-care chapter of the International Classification of Functioning in Disability [30] were assessed using items developed by the team, to evaluate independence in drinking from a cup, toileting and dressing (classified as unable, needs hands on assistance, needs occasional contact/assistance, needs verbal prompts, can do independently). Similar items for dressing and undressing were used to generate a composite dressing variable where 1 indicated any participation in dressing or undressing and 0 indicated no participation in dressing and undressing. Parents with a child with severe developmental impairments have described how hand use to enable some use of a touchscreen could be meaningful [31] in enabling reading and problem-solving skills as described in the Learning and Applying Knowledge chapter of the International Classification of Functioning, Disability and Health [30] This prompted us to generate a similarly structured item to evaluate use of a touchscreen.

### Statistical analysis

Data were reviewed for outliers where possible compared with other data in the dataset. The R package *rpart* (R Foundation for Statistical Computing, Vienna, Austria) was used to build Classification and Regression Tree (CART) analysis. The algorithm begins by identifying the predictor variable that, with an appropriate cut-off value, best splits the sample into two subgroups or nodes. The goal is to optimize model fit by minimizing within-group variability (error sum of squares) of the outcome variable. This process continues iteratively, with each node being split further until the subgroups reach a minimum size (set at 5 in this study) or no further improvement in model fit can be achieved. To prevent overfitting, a ‘pruning’ process is employed using cross-validation. Specifically, we used 10-fold cross-validation, which generates 10 non-overlapping test samples from the dataset and evaluates the average model fit across these samples for a series of trees of increasing complexity. The final model is the simplest tree that satisfies the pre-specified criterion, which ensures its fit is within one standard error of the tree with the best cross-validated performance. In addition, the CART algorithm used the standard surrogate variable method to account for missing data, If, at any stage of growing the tree, a variable involved in the primary split is missing for a group of subjects, another variable which best predicts the primary split variable is identified and subjects are assigned to one or other subgroup according to the value of the surrogate variable. In this way, no subjects are excluded from the analysis due to missingness. A variable importance plot was generated for each tree, ranking the predictor variables by their contribution to improving model fit across all potential splits whether or not the variable is used. Regression trees were constructed for the QI-Disability total score, as well as for each of the six domains separately.

## Results

### Sample characteristics

Data for 242 (94.5%) of the 256 individuals age 2 years of older who commenced the survey were analysed. The median age was 8.8 (interquartile range 4.6–14.9, range 2–50) years; 134 (55.4%) were female. Twenty-five gene causes were identified in 92.1% of individuals. The majority of respondents were the mother of the individual. Characteristics of the sample are presented in Table 1. Table 2 presents descriptive statistics for the QOL and independent variables in the regression tree models. The majority were diagnosed with epilepsy (63.6%; an additional 10.7% reported a history of seizures without a diagnosis of epilepsy), approximately one third (*n* = 82, 33.9%) were prescribed more than 2 anti-seizure medications, approximately one quarter were exclusively fed enterally (*n* = 67, 27.7%) and a small minority was able to manage dressing, toileting and drinking independently. Thirty-three (13.6%) could use a touchscreen independently. DP-4 scores were right skewed for each domain with median scores ranging from 4 in the communication domain to 7 in the social-emotional domain (Table 2). The proportions of individuals who scored 0 for each DP-4 domain ranged from 2.0% to 15.2% – Cognition: 4/203, 2.0%; Communication: 16/203, 7.9%; Social-emotional: 10/207, 4.8%; Physical: 32/210, 15.2%; Adaptive behaviour: 11/206, 5.3%.

**Table 1:** Description of demographic, affected genes and clinical features for study subjects (*n* = 242)

Variable	Level	*N* (%)
Respondent [40 (16%) missing responses]	Mother	203 (96%)
	Father	9 (4%)
Child age (years)	Younger than 5	69 (28.5%)
	5 to 12	91 (37.6%)
	12 to 18	41 (16.9%)
	18 and older	41 (16.9%)
Child sex	Female	134 (55.4%)
	Male	108 (44.6%)
Gene	*SHANK3*	45 (18.6%)
	*SCN8A*	32 (13.2%)
	*FOXG1*	25 (10.3%)
	*SCN2A*	20 (8.3%)
	*ASXL1*	17 (7.0%)
	*ASXL3*	16 (6.6%)
	*STXBP1*	11 (4.5%)
	*DUP15q*	7 (2.9%)
	*KCNT1*	6 (2.5%)
	Other (16 genes, < 5 cases)	44 (18.2%)
	Unknown or no genetic diagnosis	19 (7.9%)
Country/Region	North America	204 (81.6%)
	Western Europe	16 (6.4%)
	Australia/New Zealand	10 (4.1%)
	United Kingdom	8 (3.2%)
	Other	3 (1.2%)
*Other neurological diagnoses/symptoms*		
Intellectual Developmental Disorder	None	5 (2.1%)
	Diagnosed	165 (68.2%)
	Suspected	23 (9.5%)
	Missing	49 (20.2%)
Autism Spectrum Disorder	None	98 (40.5%)
	Diagnosed	92 (38.0%)
	Suspected	52 (21.5%)
Cortical Visual Impairment	None	133 (55.0%)
	Diagnosed	77 (31.8%)
	Suspected	31 (12.8%)
	Missing	1 (0.4%)

**Table 2:** Descriptive statistics for quality of life scores and the independent variables used in the regression trees (*n* = 242)

Dependent variable			Mean (SD)
QI-Disability (/100)		Total score	60.2 (14.1)
		Physical health domain	70.1 (14.4)
		Positive emotion domain	72.3 (20.8)
		Negative emotion domain	64.7 (20.4)
		Social interaction domain	57.2 (23.4)
		Leisure domain	63.2 (23.6)
		Independence domain	34.0 (25.6)

Independent variables			Median (range) or n (%)
Medical variables	Seizures	None	62 (25.6%)
		Some seizures, no epilepsy diagnosis	26 (10.7%)
		Diagnosed epilepsy	154 (63.6%)
	Number of Antiseizure Medications	None	85 (35.1%)
		1	39 (16.1%)
		2	36 (14.9%)
		3	28 (11.6%)
		4 or more	54 (22.3%)
	Movement disorder^a^	No	170 (70.2%)
		Yes	72 (29.8%)
	Altered muscle tone^b^	No	34 (14.0%)
		Yes	208 (86.0%)
	Looks and pay attention to objects/faces	Rarely/never	19 (7.9%)
		Sometimes	79 (32.6%)
		Usually/always	144 (59.5%)
Neurodevelopmental and Functional Ability Variables	Developmental Profile-4	Physical domain (/37)	5 (0–30)
		Cognitive domain (/42)	6 (0–37)
		Social-emotional domain (/36)	7 (0–30)
		Communication domain (/34)	5 (0–27)
		Adaptive behaviour domain (/41)	6 (0–29)
	Eating and Drinking Ability Classification System (modified)	Eats/drinks safely	20 (8.3%)
		Eats/drinks safely some mess	58 (24.0%)
		Eats/drinks some limitations	74 (30.6%)
		Eats/ drinks significant limitations	23 (9.5%)
		G-tube only	67 (27.7%)
	Communication Function Classification System (modified)	Communicates well	7 (2.9%)
		Communicates well with known	46 (19.0%)
		Inconsistently/seldom communicates	188 (77.7%)
	Spoken words	No words	175 (72.3%)
		1–5 words	36 (14.9%)
		> 5 words	6 (2.5%)
	Walking	Unable	127 (52.5%)
		Assisted	27 (11.2%)
		Independent	87 (36.0%)
	Grasping objects	Unable to grasp/hold object	35 (14.5%)
		Unable to grasp but can hold object	47 (19.4%)
		Grasps with palm	73 (30.2%)
		Grasps with pincer	86 (35.5%)
Everyday Activities of Daily Living:	Dressing	No participation	154 (63.6%)
		Any participation	88 (36.4%)
	Drinking from a cup	Unable	133 (55.0%)
		Needs hands on assistance	43 (17.8%)
		Needs occasional hands-on assistance	19 (7.9%)
		Needs verbal prompts	10 (4.1%)
		Can do independently	23 (9.5%)
	Using a touchscreen	Unable	99 (40.9%)
		Needs hands on assistance	62 (25.6%)
		Needs occasional hands-on assistance	23 (9.5%)
		Needs verbal prompts	13 (5.4%)
		Can do independently	33 (13.6%)
	Toileting	Unable	173 (71.5%)
		Needs hands on assistance	41 (16.9%)
		Needs occasional hands-on assistance	8 (3.3%)
		Needs verbal prompts	8 (3.3%)
		Can do independently	4 (1.7%)

^a^ Movement disorder included descriptions of chorea, athetosis, ataxia, tremor or stereotypies

^b^ Altered muscle tone included descriptions of hypertonia or hypotonia

### Total quality of life score

**Fig. 1:**
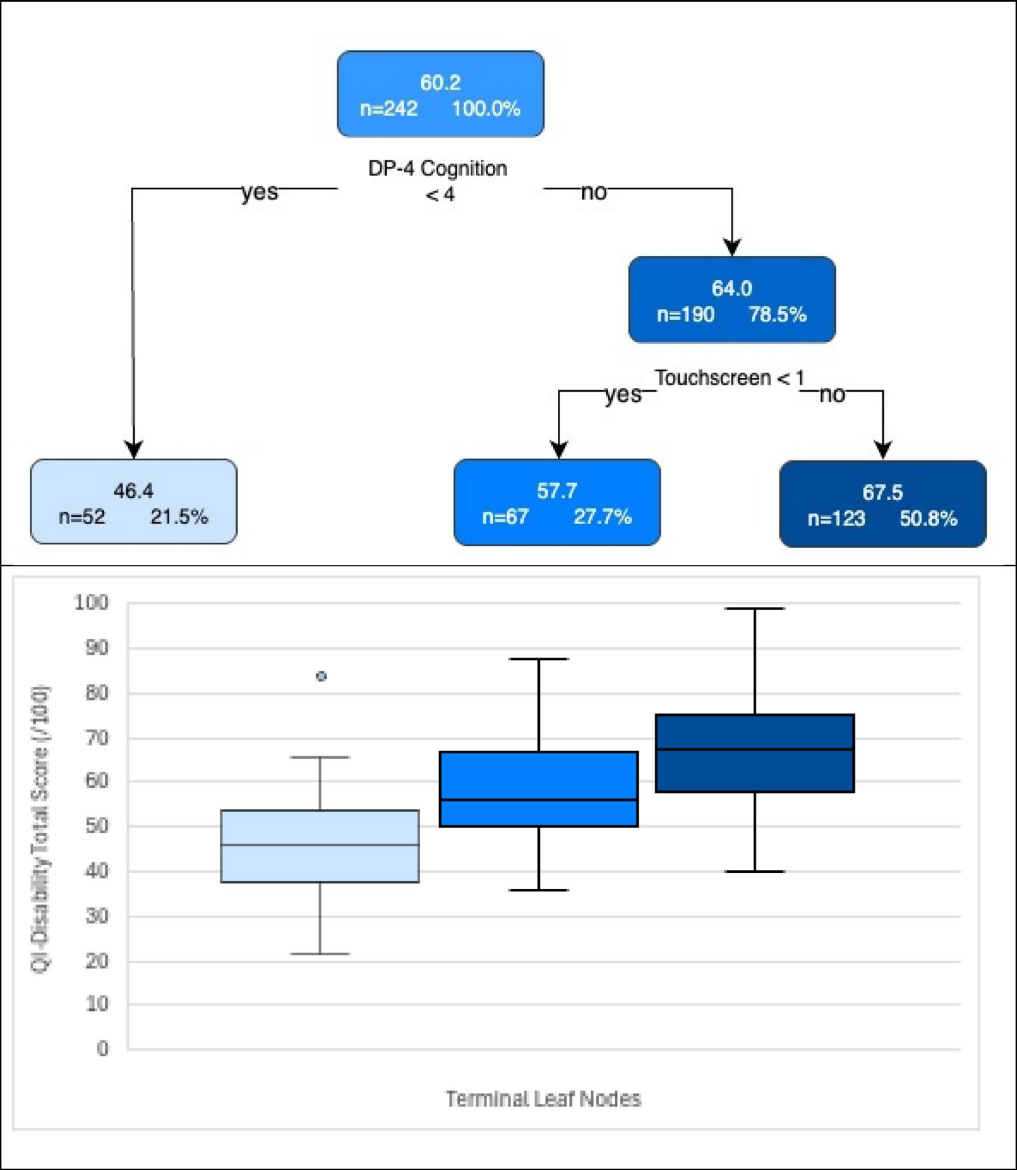
Regression tree for Quality of Life Inventory-Disability (QI-Disability) total score shows the mean total quality of life (QOL) score and n (%) for each node. Boxplots show distribution of total QOL score within each terminal node. For interpretation of DP-4 raw cognition scores, a score of 4 has an age equivalent score of a 4–5 month-level

**Fig. 2:**
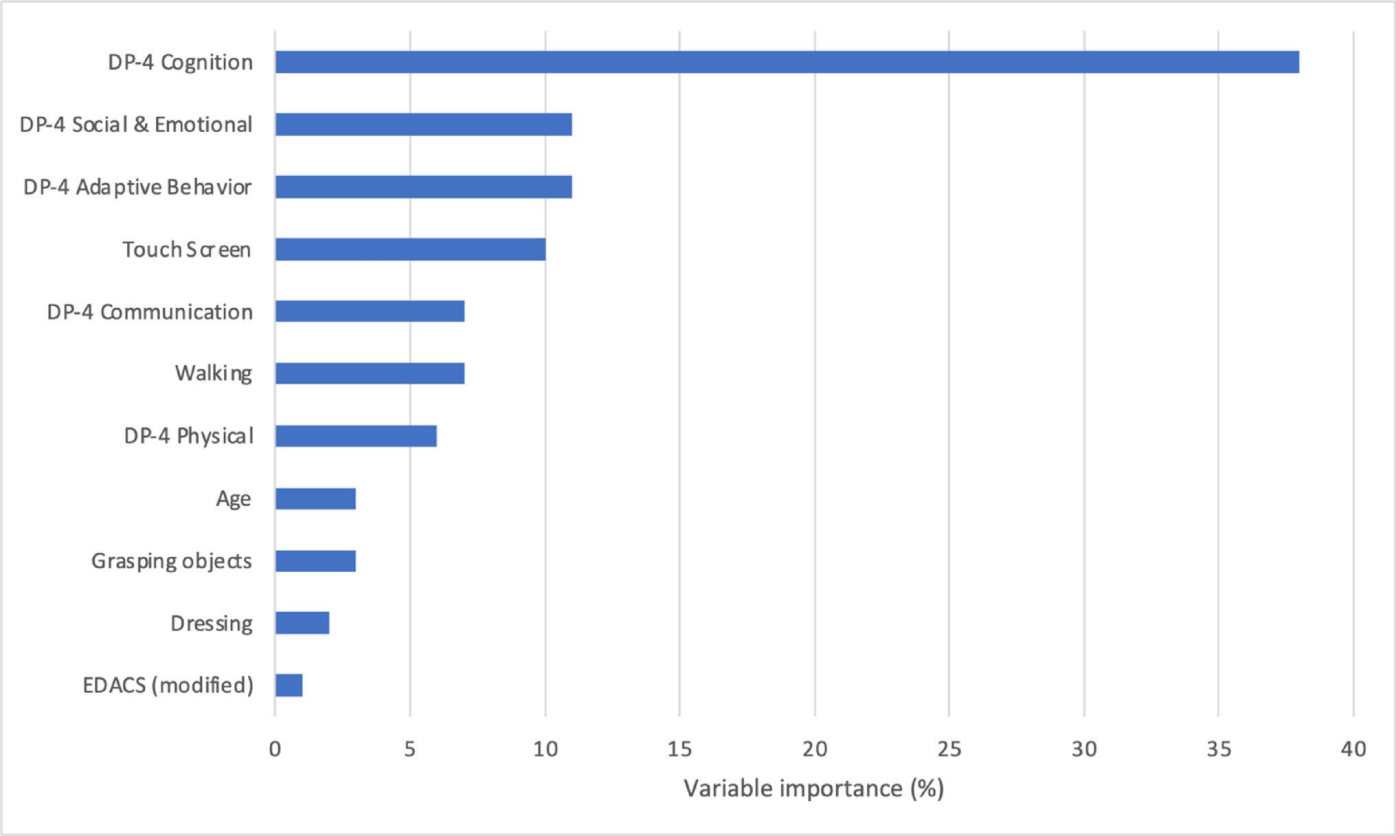
Variables in order of importance in splitting the sample by total Quality of Life Inventory-Disability score. The bar graph indicates the variance reduction contribution of variables in order in generating the regression tree (before pruning). EDACS, Eating and Drinking Ability Classification System (modified)

**Fig. 3:**
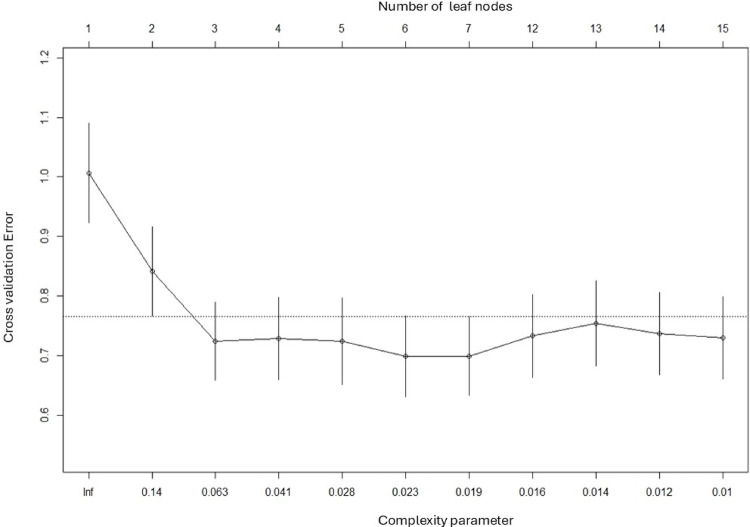
Cross validation errors (mean residual variance relative to simplest tree) for total Quality of Life Inventory-Disability score score trees of increasing complexity. The dotted line indicates the upper standard error limit of the best performing tree. Pruning selected the least complex tree (3 leaf nodes) with a cross-validation error below this limit

**Fig. 4:**
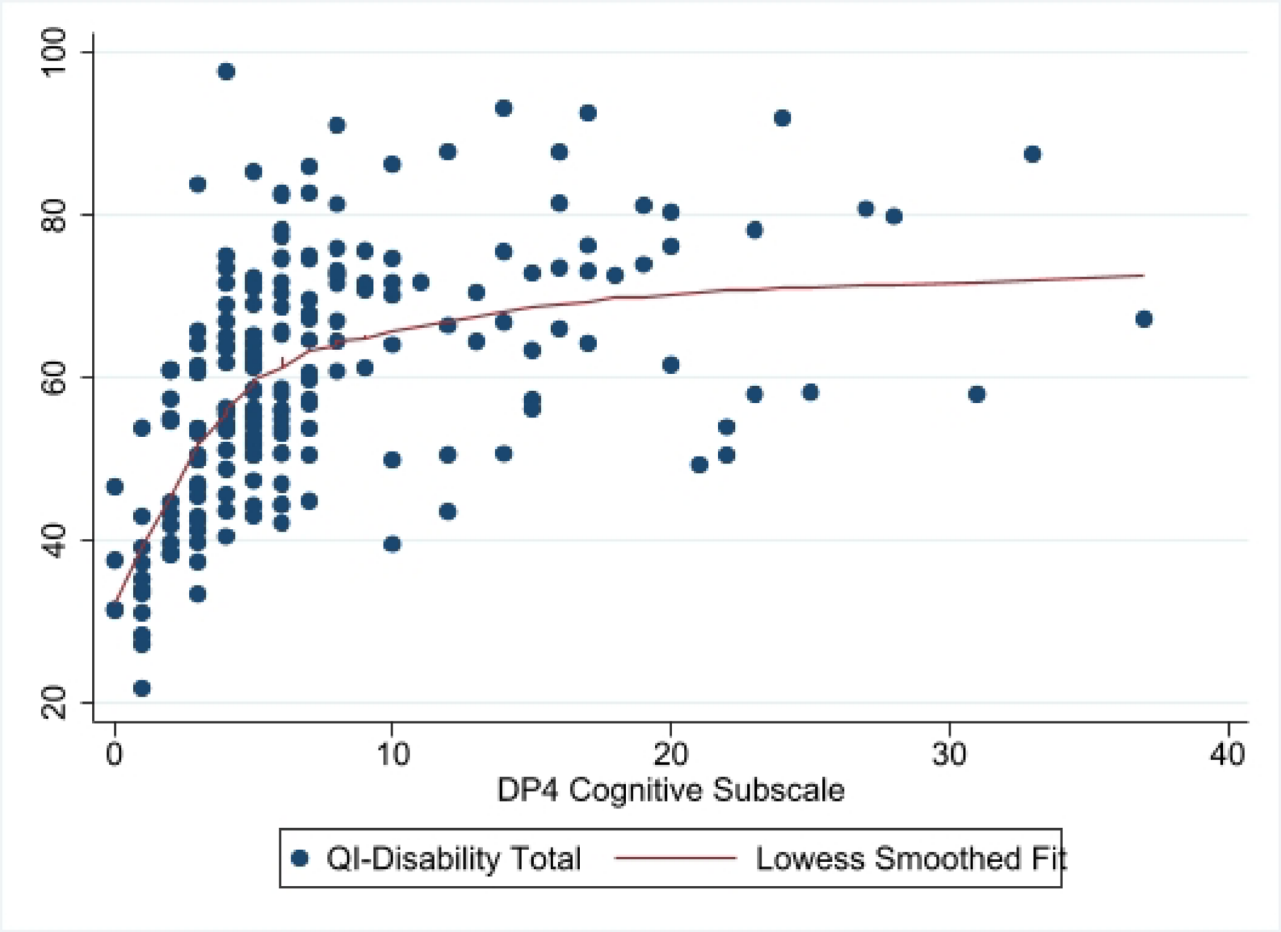
Scatterplot showing total quality of life scores and DP-4 cognition scores for each participant

The mean (SD) total QI-disability score was 60.2 (14.1) and the final tree is shown in Fig. 1. The first split, corresponding to the variable which explains most of the variance in the total QOL score, was based on the cognition domain where scores < 4 (developmental age of 4–5 months) were associated with lower QOL scores. A further split was based on child participation when using a touchscreen. The terminal subgroup or leaf with the highest QOL scores (*n* = 123, mean = 67.5) comprised children with higher ( > = 4) cognition scores and who participated to some extent when using a touchscreen. The subgroup with the lowest QOL scores (*n* = 52, mean = 46.4) contained those children with low (< 4) cognition scores. The most important variables for all potential splits are shown in Fig. 2, the cross-validation errors for trees of increasing complexity are shown in Fig. 3 to indicate how the final tree with 3 leaf nodes was selected after pruning, and a scatterplot of total QOL and cognition scores is shown in Fig. 4.

**Fig. 5:**
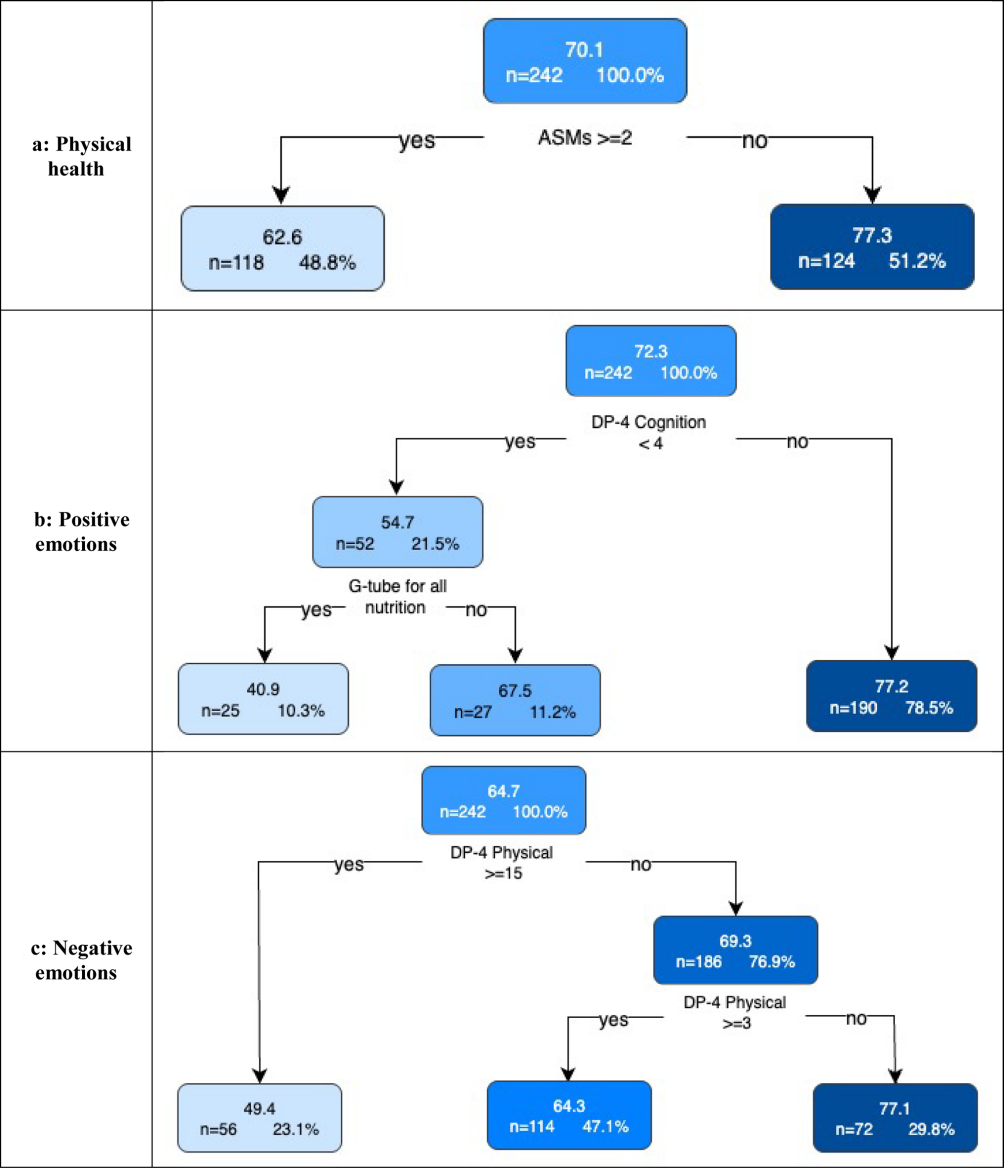

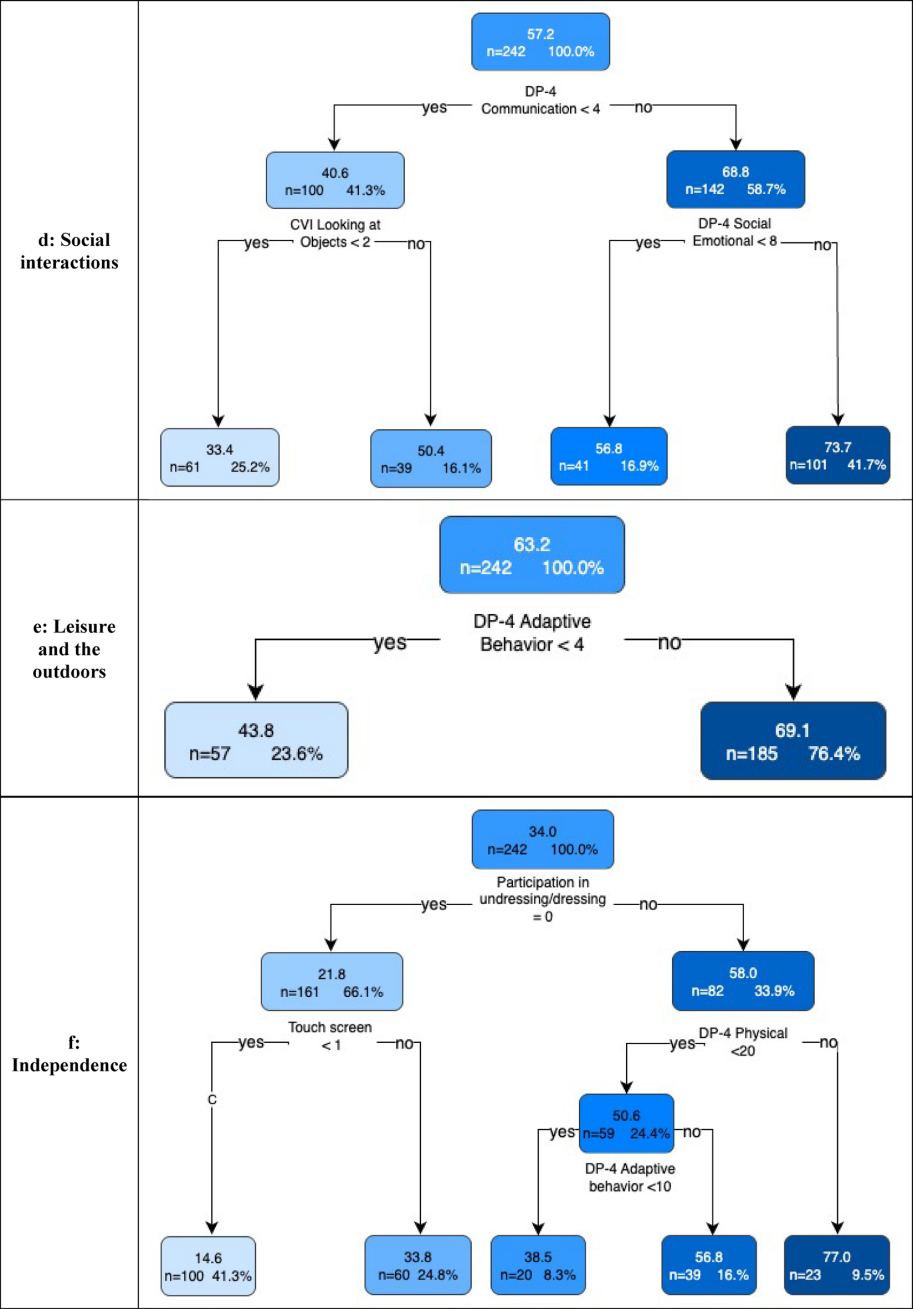
Regression trees for each quality of life domain. The first number within a leaf is the mean domain score for that subgroup of individuals, followed by the number (%) in the group. For interpretation and in order from top to bottom of the figure, a cognition raw score of 4 has an age equivalent of 4–5 months; physical raw score of 15 has an age equivalent score of 18–19 months; a physical raw score of 3 has an age equivalent score of 4–5 months; a communication raw score of 4 has an age equivalent of 6–7 months; a social-emotional raw score of 8 has an age equivalent score of 8–9 months; an adaptive raw score of 4 has an age equivalent score of 4–5 months; a physical raw score of 20 has an age equivalent score of 24–27 months; and an adaptive raw score of 10 has an age equivalent score of 16–17 months. ASM: Anti-seizure medication

### Quality of life domain scores

#### Physical health domain

The mean Physical Health domain score was 70.1. The number of anti-seizure medications (ASMs) was the only predictor variable involved in the final regression tree. Children taking two or more ASMs had the lowest domain scores (*n* = 118, mean = 62.6) while those taking no or one ASM had the highest domain scores (*n* = 124, mean = 77.3) (Fig. 5a).

### Positive emotions domain

The mean Positive Emotions domain score was 72.3. The primary split was based on the cognition domain with lower (< 4) scores associated with poorer QOL. The leaf node with the highest Positive Emotions domain scores (*n* = 190, mean = 77.2) contained children with higher ( > = 4) cognition scores while the leaf node with the lowest scores (*n* = 25, mean = 40.9) contained children with lower (< 4) cognition scores who were fully G-tube fed. (Fig. 5b).

### Negative emotions domain

The mean Negative Emotions domain score was 64.7. The DP4 physical domain was the only predictor variable involved in the final regression tree. Those children with higher ( > = 15; developmental age of 18–19 months) physical scores had lower QOL scores (*n* = 56, mean = 49.4) while those with lower (< 15) physical scores had higher QOL scores (*n* = 186, mean = 69.3), including a further subgroup with lower QOL scores for children with very low physical scores (< 3; developmental age of 4–5 months) (Fig. 5c).

### Social interactions domain

The mean Social Interactions domain score was 57.2 and the primary split was based on the DP-4 communication score with higher ( > = 4; developmental age of 6–7-months) scores having better QOL. Further splits involved CVI and the DP-4 social-emotional score. The leaf node with the highest Social Interactions domain scores (*n* = 101, mean = 73.7) comprised children with higher ( < = 4) communication scores and higher ( > = 8; developmental age of 8–9 months) social-emotional scores. The leaf node with the lowest Social Interactions domain scores (*n* = 61, mean = 34.3) contained children with low (< 4) communication scores and who never or inconsistently looked at objects or faces (Fig. 5d).

### Leisure and the outdoors domain

The overall mean Leisure domain score was 63.2. The DP4 adaptive behavior domain was the only predictor variable involved in the final regression tree. Children with higher ( > = 4; developmental age of 4–5 months) adaptive behavior scores had higher Leisure domain scores (*n* = 185, mean = 76.4) while those with lower (> 4) Adaptive Behavior scores had lower QOL scores (*n* = 57, mean = 43.8) (Fig. 5e).

### Independence domain

The overall mean Independence domain score was 34.0. The primary split was based on child participation in dressing. Further splits were based on the DP-4 physical and adaptive behavior domains and ability to use a touchscreen. The leaf node with the highest Independence domain scores (*n* = 23, mean = 77.0) contained children who participated in dressing and had higher ( > = 20; developmental age of 24–27 months) physical domain scores. The leaf node with the lowest scores (*n* = 100, mean = 14.8) included children unable to participate in dressing or when using a touchscreen. (Fig. 5f).

## Discussion

Using data driven CART analysis, cognitive skill measured by raw scores on the DP-4 played a central role in differentiating groups with higher and lower QOL scores for Total scores and Positive Emotions domain scores. Additionally, communication skills contributed to Social Interaction domain scores and physical skills to Negative Emotions domain scores. The number of ASMs contributed to the Physical Health score, DP-4 adaptive behavior scores to Leisure, and specific activities of daily living, such as dressing and using a touchscreen, contributed to Total and Independence domain scores.

The mean total QOL score in this cohort was 60.2 out of 100. This score is comparable to mean QI-Disability scores reported in other DEE studies, including *SCN8A* (50.5) [32], *CDKL5* deficiency disorder (60.3) [15] and combined DEE samples (including *SCN2A*, *SLC6A1*,* SCN1A*, 61.7) [20], and consistent also with findings of low QOL scores, representing low QOL, for Dravet syndrome using the EQ-5D [14] and Pediatric Quality of Life Inventory (PedsQL) [33] measures. The mean scores in the current study are comparatively lower than studies examining QOL with QI-Disability in samples of individuals with intellectual disability without a DEE [19]. While QOL scores vary across intellectual disability groups, they are consistently lower in DEE populations, likely due to the combined impacts of difficult to manage seizures, severe functional limitations and other neurological conditions.

Cognition distinguished between higher and lower QOL scores in the Total and Positive Emotions domain scores. Notably, the cut-point of four (i.e., age equivalent of 4 to 5 months) out of a total possible score of 41 highlights the severe impairments experienced by those with the lowest QOL. The DP-4 cognition scale measures early development of skills critical for intellectual and academic functioning. On the lower end of the scale, items focus on concepts related to alertness, a foundational aspect of cognition, which includes behaviours indicative of interaction with the environment [34]. The items slightly below the threshold assess basic visual, auditory, and emotional responsiveness to the environment. The items slightly higher than this threshold describe abilities such as sustained awareness of objects, object/person identification, object permanence and functional object use, each illustrating more responsiveness and greater interaction and engagement with the environment. Alertness has been identified as a crucial component for the conscious processing of stimuli, thereby facilitating learning and development [35–37]. This highlights the importance of assessing and supporting alertness in individuals with DEE and other severe neurodevelopmental encephalopathies [37]. Our findings reinforce the value of promoting alertness, potentially through strategies for better seizure and sleep management. Previous work has shown that there are specific therapeutic strategies that may be helpful in promoting alertness in individuals with profound intellectual and multiple disabilities, including use of multi-sensory stimuli [38, 39] and wait time to support initiation of interaction [36]. Complimenting alertness and responsiveness, abilities to participate in activities using a touchscreen could enable activities that are fun and entertaining, avenues for alternative communication, and opportunities for reading and problem solving, enabling richer participation in daily activities [30] and which could contribute to QOL.

The concepts of cognition and receptive communication are intertwined with functioning at this level. In our previous qualitative analysis of parent-reported meaningful change in functional abilities [18], better understanding and following instructions were identified as common meaningful change codes for receptive communication. These concepts are also reflected on the DP-4 cognitive scale. Indeed, the DP-4 manual cites that there could have been a sound theoretical argument made for certain items to appear on either the cognitive or communication scale [23]. Promoting understanding through strategies to build fundamental communication and cognition skills could lead to improvements in QOL. Furthermore, the CART model’s leaf node with the highest total QOL score included individuals who were independent or could participate actively in activities with a touchscreen, bringing potential for learning, entertainment and communication with others.

In the Physical Health domain, the number of ASMs was the most important variable distinguishing higher and lower scores. Drug resistant seizures are characterised by failure to achieve sustained seizure freedom after trials of at least two appropriate and tolerated antiseizure medications [40], possible for many in the current study. Multiple ASM use in the present study emerged as a significant factor influencing QOL, as for CDKL5 deficiency disorder [41], Dravet and Lennox-Gastaut syndromes [42], and SCN8A [32]. Seizures and medication-related side effects such as fatigue, cognitive impairment and behavioural changes impact the wellbeing of individuals with DEEs [43], emphasising the importance of how seizures are managed in improving QOL.

The DP-4 physical score was the most important variable to account for variance in Negative Emotions domain scores with a cut-point raw score of 15 (i.e., age-equivalent of 18 to 19 months). This suggests a complex relationship between physical functioning and emotional wellbeing. Greater motor abilities are typically indicative of less severe overall impairment but may enable individuals to express a broader range of challenging behaviours, such as frustration, anger and other negative emotions [44]. In contrast, individuals with more profound physical limitations may have fewer means to communicate their emotional discomfort, potentially leading to an underestimation of negative emotional experiences. This suggests the importance of considering expressive capacity when interpreting emotional wellbeing in this population and challenges understanding of mental health if functional impairments are severe. A meta-analysis of general populations of children with intellectual disability found similar prevalence of elevated mental health symptoms across different severities of intellectual disability [45]. A further meta-analysis of populations of children with different neurogenetic conditions found different prevalences of mental health symptoms for different conditions [46] although data for conditions with severe functional impairments were not available in this analysis. We recommend that future studies investigate the complex relationships between functional impairments, mental health, behaviors and quality of life across the spectrum of intellectual disability.

A more complex pattern of influencing factors emerged for the Social Interactions domain, reflecting the multifaceted nature of social interactions, which not only involve communication skills but also the ability to connect and engage with others on social and emotional levels [47]. Risk for CVI (i.e., ability to see an object/face and pay attention to it or not) was also an important factor. Impairments in visual processing can make it difficult to interpret non-verbal cues [48] such as facial expressions and body language and highlights the need for tailored supports and adaptations to enhance social interactions for individuals with DEEs and other severe neurodevelopmental encephalopathies who experience poor vision. Similarly, the Independence domain score was influenced by a combination of factors, including dressing abilities, use of touchscreen, adaptive behaviours and physical functioning. Active engagement and participation in activities that are enjoyable and satisfying to the individual, particularly those that foster autonomy, can promote QOL.

The CART analysis highlighted nuanced and individual differences where small score differences had important associations with QOL. For example, the scatter plot shows that the greatest impact on QOL came from small, yet meaningful, changes at the lower end of the cognitive ability range (Fig. 3 to Fig. 4). Further, the DP-4 domain scores and levels of participation in daily activities appeared sensitive to differences in QOL. These data indicate the importance of individual assessment for individuals with severe functional impairments to inform the delivery of targeted clinical therapies to support QOL

This study had several strengths, including a moderately large sample size of individuals with a range of genetic aetiologies, all of whom shared severe functional impairments. The presence of shared functional characteristics across participants supported the use of a transdiagnostic approach to explore factors influencing QOL. The QOL measure employed was a validated measure suitable for this population. However as with all cross-sectional studies, causal relationships cannot be established. Future longitudinal work is needed to explore causal relationships and suggest mechanisms of effect. We advertised the study and cannot report the response fraction. Additionally, this study is limited by convenience sampling which may reduce representativeness of findings. With any patient/caregiver reported survey, there are risks of recall error. While a broad range of predictor variables was included, we acknowledge the absence of data on pain, sleep, and community participation which are important predictors of QOL in other intellectual disability studies [49]. Including these variables would have enabled a richer description of participant strengths and difficulties, consistent with the International Classification of Functioning in Disability [30] and should be evaluated in future research. We acknowledge that our measures of everyday activities were designed for this study and whilst parent reviewers of the survey prior to administration viewed the questions as clear and the response options as comprehensive, validation is required. Lastly, whilst our regression tree model offers valuable insights, it could not be validated with an independent sample due to the limited overall sample size which was not large enough to split into two independent samples.

## Conclusion

The application of regression tree analysis to individuals with multiple genetic diagnoses and severe neurodevelopmental impairments highlighted associations between functional skills, participation and QOL. These distinguishing factors point to potential intervention pathways, such as promoting alertness, social engagement and participation in daily living tasks, that could be targeted to enhance QOL in individuals with severe functional impairments. Findings suggest that even small changes in skills could have important impacts. Results could be generalisable to other populations with severe neurodevelopmental impairments. Future research should explore these relationships longitudinally as well as targeted person-cantered strategies that address these functional and participation skills.
